# A Congolese community-based health program for survivors of sexual violence

**DOI:** 10.1186/1752-1505-6-6

**Published:** 2012-08-29

**Authors:** Anjalee Kohli, Maphie Tosha Makambo, Paul Ramazani, Isaya Zahiga, Biki Mbika, Octave Safari, Richard Bachunguye, Janvier Mirindi, Nancy Glass

**Affiliations:** 1 Johns Hopkins Bloomberg School of Public Health, Baltimore, MD, 21205, USA; 2 Foundation RamaLevina, Bukavu, Democratic Republic of Congo, Africa; 3 Johns Hopkins University School of Nursing and Johns Hopkins Center for Global Health, Baltimore, MD, 21205, USA

**Keywords:** Sexual violence, Gender based violence, Democratic Republic of Congo, Community based programmes, Women’s health services, Mobile health care

## Abstract

Many survivors of gender based violence (GBV) in the Democratic Republic of Congo (DRC) report barriers to access health services including, distance, cost, lack of trained providers and fear of stigma. In 2004, Foundation RamaLevina (FORAL), a Congolese health and social non-governmental organization, started a mobile health program for vulnerable women and men to address the barriers to access identified by GBV survivors and their families in rural South Kivu province, Eastern DRC. FORAL conducted a case study of the implementation of this program between July 2010-June 2011 in 6 rural villages. The case study engaged FORAL staff, partner health care providers, community leaders and survivors in developing and implementing a revised strategy with the goal of improving and sustaining health services. The case study focused on: (1) Expansion of mobile clinic services and visit schedule; (2) Clinical monitoring and evaluation system; and (3) Recognition, documentation and brief psychosocial support for symptoms suggestive of anxiety, depression and PTSD. During this period, FORAL treated 772 women of which 85% reported being survivors of sexual violence. Almost half of the women (45%) reported never receiving health services after the last sexual assault. The majority of survivors reported symptoms consistent with STI. Male partner adherence to STI treatment was low (41%). The case study demonstrated areas of strengths in FORAL’s program, including improved access to health care by survivors and their male partner, enhanced quality of health education and facilitated regular monitoring, follow-up care and referrals. In addition, three critical areas were identified by FORAL that needed further development: provision of health services to young, unmarried women in a way that reduces possibility of future stigma, engaging male partners in health education and clinical care and strengthening linkages for referral of survivors and their partners to psychosocial support and mental health services. FORAL’s model of offering health education to all community members, partnering with local providers to leverage resources and their principal of avoiding labeling the clinic as one for survivors will help women and their families in the DRC and other conflict settings to comfortably and safely access needed health care services.

## Background

Conflict affects civilian populations “physically, psychologically, economically, and socially” [1]. The Eastern Democratic Republic of Congo (DRC) has been the site of two wars involving multiple African nations and armed conflict between rebels and soldiers for the past 16 years. Several reports document the number and severity of cases of violence perpetrated against civilians, especially with a focus on sexual violence [2,3]. Although insecurity and fear related to reporting cases limits surveillance of sexual violence [4], in Eastern DRC two surveys estimated the prevalence of sexual violence against women (16-40%) and men (24%) using population-based approaches [5,6]. Between 2004 and 2008 at Panzi Hospital, a referral hospital in South Kivu province, girls between 3.5 years to women 80 years of age accessed services for sexual violence [7]. Several reports describe the brutality accompanying sexual violence including mutilation and penetration with objects [7,8]. A review of medical records from two rural NGOs and Panzi Hospital revealed that 59% of patients accessing services for sexual violence in 2006 were gang-raped [3]. Physically, the consequences of sexual violence include traumatic fistula and risk of infections including STI/HIV [7,8]. If injured during the sexual assault, survivors are more likely to experience post-traumatic stress disorder related symptoms [9]. Other mental health concerns include depression and difficulty participating in daily activities [3,10].

As demonstrated by the high infant mortality rate (114/1000) and maternal mortality ratio (1,100/100,000), poor health outcomes continue to be an important issue throughout the DRC [1]. The 2011 UNDP Human Development Report ranked the DRC last amongst 187 countries in a composite measure of human development [11]. While the health system was deficient before the conflict [12], violence, destruction, limited resources and displacement has further limited the ability of the health system to function well and provide needed services [13].

In South and North Kivu Provinces and Ituri District, a household survey (2010) conducted in accessible and secure villages found that 67% of the population lacked adequate access to general health care [6]. Delayed access to health services for survivors of gender based violence (GBV) can result in serious health issues [14,15]. Sexual violence survivors accessing services from Panzi Hospital in 2006 reported a 16-month average delay between assault and health care [7]. Reasons for delay include fear of stigma if sexual assault is discovered by husband and/or family, cost of transport and care, lack of knowledge about services, insecurity and under-resourced facilities [8,16].

Mobile health clinics have been used in different settings to reach vulnerable populations, increase access to services, provide culturally appropriate and holistic services and improve coverage of interventions such as HIV/AIDS testing in Malawi [17], maternal health care in The Gambia [18], family planning [19] and acute disorders, such as respiratory illness and gastrointestinal disorders in Ethiopia [20]. Studies in Malawi and Ethiopia on the provision of mobile health services to supplement services provided in rural health centers indicated that mobile health services often reach rural populations that do not access services but require strong partnerships with local leaders and service providers to be effective [17,20]. Foundation RamaLevina (FORAL) a Congolese led mobile health and social program in rural Walungu Territory, provides services for vulnerable women including survivors of GBV and their male partners. The objective of this case study is to examine the revised mobile care strategy that was developed by FORAL staff, local HCPs, leaders and recipients of health services to address issues of quality and sustainability. The revised mobile health strategy, implemented in 6 villages of Walungu Territory over the course of one year (July 2010 – June 2011), had three primary components:

(1) Expansion of mobile clinic services and visit schedule;

(2) Clinical monitoring and evaluation system; and

(3) Recognition, documentation and brief psychosocial support for symptoms suggestive of anxiety, depression and PTSD.

Following a description of the mobile health program, each component of the revised strategy is described in more detail below.

### Case description

#### Development of a Congolese response to GBV

Paul Ramazani and Dr. Maphie Tosha Makambo established FORAL, a Congolese NGO, in 2004 to develop and implement a health care response for survivors of GBV in their community. FORAL has based its program on their belief in the ability of the Congolese people to find strategies and solutions, with appropriate support and resources, to respond to community challenges. In 2006, FORAL conducted a situational assessment of the health needs of survivors in Walungu Territory. FORAL chose the Walungu Territory due to the proximity to Bukavu to facilitate access, the high level of violence villagers experience during the war and the need for health, economic and social services to assist in rebuilding the area. The situational assessment included interviewing local leaders, health care providers (HCP), community members and survivors of GBV on their priorities for local programs and resources. The findings from the situational assessment indicated that priorities for programs focused on two main areas: 1) improved access to quality health services with skilled HCP, who had specific training in care for GBV survivors; and 2) increased opportunities for GBV survivors to be contribute economically to their family and community. In partnership with existing health services in the area, FORAL initiated a free mobile health program for survivors and other vulnerable women and girls and, later, expanded services to include socio-economic and reintegration services for survivors and their families and awareness education to reduce stigma in the community.

### FORAL’s Mobile health program

The targeted areas for FORAL’s mobile health services in Walungu Territory include the village centers: Cagala, Ikoma, Izege, Kaniola, Murhali and Mwirama. FORAL’s program engages community members through partnerships with community health workers (CHW) – respected individuals who are known for supporting neighbors to deal with loss of family, rejection and stigma due to sexual violence. CHW build relationships with survivors and educate them about available health services. Other FORAL team members include 2 physicians, a nurse, a coordinator and a logistician. Clinic activities begin with health education led by the FORAL physician and the health center nurse and is offered to all village members in the local language. The educational topics focus on STI/HIV, hygiene and engaging male partners in health care. These sessions provide an opportunity for FORAL team members to form relationships with community members through exchanging information and answering questions. When local community leaders and partner NGOs expressed an interest in attending the education sessions, they were invited to participate and exchange ideas.

All FORAL staff received training in the provision of ethical, compassionate and competent care for GBV survivors. To reduce potential stigma associated with GBV, FORAL does not limit health services only to GBV survivors. However, the majority of women who seek care reported a history of GBV, often sexual violence related to a conflict situation. Through meetings with the Director of Medicine for the Health Zone and Administrators of the villages and Territory, FORAL formed partnerships with government and church sponsored primary health centers (PHC) and hospitals in Walungu and neighboring Kaniola Health Zones. These partnerships were critical to demonstrating respect to the existing expertise, ensuring services were appropriate and not redundant and gaining support from local leaders and HCP. This also allowed for capacity building between FORAL and existing health systems in the area, resource sharing and discussions about sustainability. Depending on space, FORAL mobile clinic services were provided within a dedicated space in the PHC or in a tent located just outside the PHC; this ensured that visits to the mobile clinic were seen as part of normal health services, not services only for raped women.

Initially FORAL implemented the clinic in each of the 6 village centers on a rotating basis mostly dictated by their ability to access donations from Congolese friends, family, churches and businesses and to obtain medication and transport. Due to insecurity in the targeted area, FORAL providers arrived early in the morning but needed to be on the road back to Bukavu before 5 pm as to make it back prior to sunset. The daily mobile clinic activities included clinic set-up and the interactive health education session followed by individual health care services for women. Between 30-60 women were seen at the clinic during the day, meeting about 1/3 of the requests by the community. CHW assisted providers in prioritizing services based on women’s report of symptoms. With consent from the patient, several tests were conducted on blood and urine including HIV, syphilis and hepatitis B. Laboratory tests were conducted on-site or in Bukavu at a local technical school. FORAL escorted and referred patients with more serious health complications including HIV to the reference hospital in the territory or Bukavu although funding remained a barrier to access specialty health services. Following the PHC data management system, clinician’s recorded health information on the patients’ health card and returned it to the patient. At the end of each clinic day, a team meeting was held to allow for sharing information as appropriate and resolving problems. These meetings ensured that the entire team participated in improving clinic services. After three years of implementing mobile health services, FORAL engaged partner health service providers, community leaders and survivors in a review and revision of the mobile health care strategy with the goal of improving and sustaining health services in the targeted villages and beyond. To assess the results of the revised strategy, this case study focused on understanding the key indicators collected from the clinic monitoring and evaluation system including number of patients receiving treatment for the first time after experience of violence, partner treatment and STI cases. In addition, the team sought feedback from service recipients, HCP and FORAL team members charged with implementing the revised strategy.

### Implementation of revised mobile clinic program

The revised mobile health service strategy had three primary components. Below, each is described in detail followed by descriptive results from the mobile health services.

#### Expansion of the mobile clinic services and visit schedule

The revised strategy involves a full rotation (4 visits in one month) of mobile health services for the village members before initiating health services in the next village. Previously, FORAL provided clinical services once in each village center before returning for follow-up care. The 4 visits during the month were strategically developed to (1) allow for relationship building between the provider, GBV survivor or other vulnerable women, and their male partner and, (2) ensure retention of patients and improve quality of care (assessment of treatment outcomes, explanation of test results) at follow-up. FORAL’s mobile clinic services are located at the PHC, typically within 30 – 90 minutes walking distance from the targeted villages. The CHW spreads the word throughout the village about the mobile clinic visit schedule and encourages women and others to attend if they need care. For women who have male partners and report symptoms consistent with STIs, FORAL providers discuss with the woman about the need for treatment for self and partner and provide a packet of antibiotics for her and her partner. FORAL physicians and nurse discuss with the women the importance of having their male partner take the medications regardless of symptoms and the strategies they may use to have their partner join them for the follow-up visit.

In follow-up visits, physicians asked patients whether they completed their course of antibiotics, and, if appropriate, whether their male partner took the treatment that was sent home. Not all patients were given follow-up visits; appointments were determined based on diagnosis at initial assessment, treatments provided and availability of test results. For example, follow-up visits 2 and 3 focused on care for patients with unresolved STI or other illnesses after completing one course of antibiotics. Patients with complex issues or those that were not responding to treatment were referred to partner health organizations that have resources for specialty care. Table 1 summarizes the activities for each mobile clinic visit.

**Table 1:** Mobile health care monthly service plan

**Visit**	**Activities**	**Rationale for visiting on this day**
**Visit 1, Day 1**	Education session	First visit
	*New Patients:* rapport building, patient history, chief compliant and exam, collection of samples for laboratory test, treatment, follow-up appointment for those given treatment or samples taken for lab test	Patients in need of health services identified in partnership with CHW
**Follow-up Visit 1, Day 10**	Education session	Completion of a course of antibiotics typically requires 7-10 days.
	*New Patients:* see Visit 1	
	*Follow-up visit:* evaluation of treatment (adherence, symptoms), take culture, revise treatment if needed, patient counseling and education on reproductive health and mental health	
**Follow-up visit 2, Day 14**	Education session	Culture results are available after three days
	*New Patients:* see Day 1	
	*Follow-up visit:* assess health status, provide/discuss test results, treatment, counseling and referral.	*this clinic visit is for those women who have unresolved symptoms of STI after Follow-up Visit 1.
**Follow-up visit 3, Day 24**	Education session	Treatment completed after 10 days
	*New Patients:* see Day 1	
	*Follow-up visit:* evaluate treatment and patient health, refer case to health facility if problems persist	*this clinic visit is for those women who received treatment on Follow-up Visit 2.

#### Clinic monitoring and evaluation system

The clinical monitoring and evaluation system was developed in 2010 following discussions with CHW, local HCP and academic partners. Previously, FORAL lacked monitoring data as the staff returned the health cards to the patient. The newly implemented standardized clinical forms have four sections that are progressively completed with each follow-up visit. The first section included an assessment of patient history, demographics, experience of sexual violence, receipt of medical care after the assault(s), clinical exam, symptoms indicative of physical and mental health problems and planned treatment. Minimizing the burden of documentation for both patient and clinic staff was a priority due to the limited duration of the clinic (6 hours daily), number of patients (30-60), travel time and home responsibilities of patients. Physicians documented the chief complaints and observed and discussed health problems (e.g., lack of eye contact, chronic pain) with the patient. The form was designed to move from general information to more sensitive issues as the provider gained the trust of the patient. Sections 2-4 of the clinical form focused on follow-up services. In all sections, the physician documented treatment and test results, with referrals for additional services as needed.

#### Recognition, documentation and brief psychosocial support for symptoms suggestive of anxiety, depression and PTSD

FORAL staff are not mental health professionals, yet they recognize the need to better understand the mental health issues of their patients. To effectively respond to mental health needs, the team focused on identifying and documenting the different types of behaviors and symptoms observed and reported by patients. FORAL physicians and nurses collaborated to conduct a brief review of symptoms of mental health distress that had been observed or described by patients or community members during their work in villages. These symptoms (listed in Table 2) included a section where patients reported symptoms (e.g., headaches, shock) and where physicians documented observations including lack of eye contact and self-care. The symptoms on the clinic tool were not taken from a validated or adapted diagnostic tool. The purpose was not for diagnosis, but, instead, to guide staff in providing psychosocial support to patients as well as identifying capacity building needs of FORAL and health care partners.

**Table 2:** Symptoms associated with mental health distress amongst survivors of sexual assault (N = 657)

	**n (%)**
***Anxiety-like symptoms***	
Anxious or troubled	196 (29.8%)
Acts panicked	70 (10.6%)
Moves to protect self	17 (2.6%)
***Depression-like symptoms***	
Sadness	288 (43.8%)
Speaks slowly	150 (22.8%)
Creates a reason for why the assault happened	137 (20.8%)
Moves slowly	105 (16.0%)
Blames self for the incident	55 (8.4%)
Loss of appetite	49 (7.4%)
Cries easily and often	8 (1.2%)
Feels hopeless about the future	4 (0.6%)
Has suicidal thoughts	2 (0.3%)
Self mutilation	0 (0%)
***PTSD-like symptoms***	
Feeling shameful	226 (34.4%)
Embarrassed	148 (22.5%)
Trouble sleeping (e.g., insomnia, nightmares)	115 (17.5%)
In shock	92 (14.0%)
Withdrawn into self	92 (14.0%)
Has flashbacks, relives the event	85 (12.9%)
Avoids people/situations	78 (11.9%)
Has trouble with memory or concentration	74 (11.3%)
Hypervigilant	25 (3.8%)
Disoriented and confused	16 (2.4%)
Loss of memory or inability to concentrate	10 (1.5%)
Does not speak	3 (0.4%)

#### Case study findings: descriptive results during one year (July 2010 – June 2011)

Between July 2010 and June 2011, FORAL treated 772 women in the 6 villages (Table 3). Of the 772 women treated, 657 (85%) reported being survivors of sexual violence. The remaining data focuses on the 657 survivors of sexual violence that received services during this period. About 72% of patients returned for their scheduled first follow-up visit, however attendance at follow-up visits dramatically drops for the second and third follow-up visits. Due to the reduced number participating in second and third follow-up visits, FORAL decided that logistically and financially it made sense to have one physician provide health care for the second and third follow-up visits and the other physician initiate mobile health services in a different village center on the same day.

**Table 3:** Demographic information of women treated by FORAL mobile clinic, July 2010 - June 2011

	**n (%)**
Total women treated	772
Survivors of sexual violence	657 (85.1%)
Total survivors treated in follow-up visit #1	476 (72.4%)
Total survivors treated in follow-up visit #2	44 (6.7%)
Total survivors treated in follow-up visit #3	2 (3.0%)
***Information on Survivors treated at FORAL Clinic:***	
**Age Groups**	
0 - < 20 years	6 (0.9%)
20 - 29 years	113 (17.2%)
30 - 39 years	146 (22.2%)
40 - 49 years	173 (26.3%)
> = 50 years	219 (33.3%)
**Marital Status**	
Single	20 (3.7%)
Married/Not polygamous	231 (43.1%)
Married/Polygamous relationship	101 (18.8%)
Separated/Rejected by Spouse	106 (19.8%)
Widow	78 (14.6%)
**Number of children**	
0 kids	21 (3.3%)
1 - 3 kids	177 (28.1%)
4 - 6 kids	267 (42.3%)
> = 7 kids	166 (26.3%)
Year of most recent sexual assault*	
Before 2006	282 (46.4%)
2006	98 (16.1%)
2007	118 (19.4%)
2008	55 (9.0%)
2009 - 2011	55 (9.0%)
**Place of most recent sexual assault**	
Home	552 (89.9%)
Field/While cultivating	20 (3.3%)
Forest	26 (4.2%)
On route/While walking	11 (1.8%)
Other	5 (0.8%)
**Ever received health care services after sexual assault**	
Yes	331 (55.2%)
No	269 (44.8%)

*It was not possible to further breakdown year of more recent sexual assault into 2009, 2010, 2011 based on the way data was recorded. This reflects self report of sexual assault amongst women accessing services.

The majority of women seeking and receiving services were older than 40 years (60%) of age. Less than 1% of those receiving services were under 20 years old. Most women were married (62%) and 20% reported being rejected/abandoned by their husband as a result of sexual violence. The majority of sexual assaults occurred in the home (90%) of the survivor and her family. Almost half of the women (45%) reported never receiving health care services after the last sexual assault.

FORAL offers all patients an HIV test (Table 4). Of the 772 women who visited the mobile clinic, 714 (93%) gave consent for an HIV test. An overall 1.8% of 714 women were positive on both the first and second confirmatory test. FORAL physicians conducted health assessments and laboratory tests as appropriate; 84% on assessment had symptoms consistent with STI (e.g. painful urination, discharge). About 40% of the survivors were diagnosed and treated with other illnesses, including malaria, urinary tract infections, intestinal parasites, and chronic pain.

**Table 4:** Diagnosis and partner treatment

	**n (%)**
**HIV Test amongst all female patients (survivors and non survivors) using clinic services**	
Total who consented to having an HIV test	714 (92.5%)
Total with a confirmed HIV positive test result	14 (1.9%)
**Symptom based diagnosis for survivors of sexual assault using clinic services**	
Symptoms similar to STI*	549 (83.6%)
Urinary infection	124 (18.9%)
Other infection (e.g., malaria, fever)	254 (38.7%)
Male partners of survivors treated for STI symptoms**	
Total number given treatment	311
Completion of full treatment	127 (40.8%)
Completion of partial treatment	34 (10.9%)
Refused treatment	24 (7.7%)
Treatment status unknown	126 (40.5%)

*Symptoms similar to STI include pain while urinating, unusual discharge, pain during sex, lower abdominal pain, itching, etc.

**Full treatment refers to women who reported that their male partners completed the full course of antibiotics, partial treatment refers to women who reported their male partners started but did not complete treatment for STI symptoms, and refused treatment refers to women who reported their male partners refused to start treatment for STI symptoms that was sent to them by FORAL physicians. Treatment status is unknown in cases where female partners did not participate in follow-up visits or were unable to report treatment status.

Forty-one percent (N = 311) of survivors reported that their male partners completed treatment and 8% reported that their partner refused treatment. Male adherence was unavailable for 41% of male partners, as the women did not attend follow-up visit or were unable to report whether their partner completed treatment. The most frequently reported or observed mental health symptoms (Table 2) included sadness (37%), shame (29%) and feeling anxious or troubled (25%).

### Discussion and evaluation

A one year review of the revised FORAL program provides detailed information on a Congolese-led mobile health program for survivors of GBV, other vulnerable women and their male partners in rural post-conflict settings that often lack access to skilled providers. In partnership with the existing Congolese health system and community leaders, FORAL’s program can reach and provide much needed care to survivors and others in need of health services. Partnerships with providers in the existing systems ensures local expertise on context and program strategies, not duplicating services and exchange of ideas and respect [17]. The expanded mobile clinic services and visit schedule allowed FORAL HCP to build relationships with village members, especially important given the sensitivity of the issues discussed, alter treatment plans as needed, distribute and discuss test results and provide referrals for appropriate care.

Discussions with CHW working in the villages confirmed provider reports of increased patient satisfaction with the revised schedule and engagement in treating male partners. The educational session at the beginning of each mobile clinic allowed the local CHW to assist FORAL providers in targeting education sessions to community concerns including those concerns that community members had but felt uncomfortable presenting. Further, these sessions supported the CHW confidence to provide village members with accurate information on STI/HIV prevention and other topics. Community members participated more actively in education sessions as evidenced by answering and posing questions to FORAL and local staff during and after the sessions. FORAL’s efforts to protect the identity of survivors of GBV, build relationships with patients and provide targeted health education contributed to patient appreciation of the compassionate, non-judgmental and high-quality care received at the FORAL mobile clinic. Other studies also emphasize the importance of targeted health education and confidential, non-judgmental care in order to provide timely and high-quality health care especially for illnesses that are associated with stigma such as HIV/STI [21-23].

The monitoring and evaluation system developed by FORAL did assist the providers and CHW with establishing appointments for follow-up in a confidential setting. Yet, travel, distance or other commitments sometimes prevented CHW from reminding patients about appointments and thus, follow-up rates were not as high expected. Providing four mobile clinics in each village during a one month period improved retention but did not resolve all the issues, as about 70% of patients returned for at least one follow-up visit. Rates of follow-up dropped dramatically on second (7%) and third (3%) follow-up visits. As with other health interventions, many patients who started to feel better with medication may not prioritize or understand the need for follow-up consultation. HCP and CHW continue to emphasize and explore ways to increase retention and appropriate referrals for ongoing follow-up care.

While summarizing the patient information was time-consuming, the new medical record system allowed FORAL to better understand their target population and treatment effectiveness. FORAL also reported having information from the medical records that allowed for effective management and planning for health clinics including purchase of appropriate medications. Centralizing services to the six villages that are easily accessible to other villages and combining second and third follow-up visits with initiation of clinic services in a different village reduced the transport cost allowing FORAL to shift resources to purchase needed clinical supplies.

Staff reported that the completion of the forms were not time-consuming, facilitated physician recall and treatment evaluation at follow-up, and ensured systematic collection of information. To facilitate future monitoring and evaluation, FORAL plans to implement a computerized data management system in partnership with Johns Hopkins University. Importantly, the medical record data showed that almost 50% of services provided were to survivors who had never received health care after sexual violence and about 84% had symptoms consistent with STI. This confirms the situational assessment, which indicated that many survivors had limited access to skilled HCP.

The medical record data also highlighted gaps in the FORAL health program. For example, during one year of mobile health services, 99% of patients accessing care were 20 years or older. Other FORAL projects identified females younger than 20 years that experienced sexual violence. The FORAL team reached out to several young adults (15-20 years old) to understand why they were not accessing services through the mobile clinics. Although the young women acknowledged a need for health care, they were concerned that while seeking care they would be seen by older women who could be their future mother-in-law. Older women may then label a younger woman as a survivor or sexually active; thereby reducing her opportunities for marriage. A study with Ugandan adolescents also found that lack of confidentiality can limit access to care [24]. FORAL will continue to work with young women to provide services in a way that reduces risk for stigma. In consultation with young women, FORAL is exploring whether and how to increase the capacity of local HCP to provide needed health care to survivors of GBV, integrate health care with socio-economic interventions targeted to young women, increase inclusion of women that have not experienced GBV or provide separate mobile health services for youth.

Treatment of male partners for STI remains a challenge. Women reported difficulty talking about STIs, medications and the reason for treatment with their husbands/male partners. Sex and reproductive health are not openly discussed between intimate partners; survivors often fear their partners will reject them if they find out they are sick. Women who do raise the issue with their partner often reported that he disregarded the information or did not understand why he needed treatment when he had no symptoms. A study in South Africa indicated that patient delivered partner medication can be used to treat STI when good communication exists between the couple [25]. Strategies to improve partner treatment may include inviting men to health education sessions [26], improving partner communication, assessing for issues of interpersonal violence and safety in the relationship and reducing stigma associated with violence exposure and STI/HIV.

Studies in conflict-affected countries point to a relationship between exposure to traumatic events, ill health without medical care, economic instability and mental health outcomes [27,28]. Symptoms associated with mental health concerns were documented based on observation by HCP and self-report. FORAL understands the importance of building capacity to provide brief counseling and appropriate referrals for psychosocial support. There were few local resources for survivors and their families; services often established by an international NGO can include a “Listening House”, similar to Western models of safe houses or support groups. However, the sustainability of Listening House model has been limited by funds and capacity. Family mediation, a local intervention provided by respected community members to resolve conflict in the family and community, may be a strategy to address psychosocial support needs of both the survivor and her partner. However, many of the family mediation programs are informal and often do not have the capacity to reach all those in need. Interventions with other conflict-affected populations suggest that use of sociotherapy to build social bonds [29], group interpersonal therapy [30] and socio-economic interventions [28] may address the psychosocial needs of conflict-affected populations. Continued work with local communities to understand options and strategies to address psychosocial needs of survivors remains important.

## Conclusions

FORAL’s mobile clinic strategy and their approach to working with local partners can inform organizations working in rural, conflict-affected areas. This case study illustrated: (1) that more frequent visits may improve provider-patient communication and relationships and allow for targeted health education, care and treatment to survivors of GBV and male partners; (2) the importance of local partnerships to avoid redundancy and increasing opportunities for leveraging and sustaining efforts; (3) the value of a monitoring and evaluation system to improve services; and (4) the need for locally relevant and sustainable psychosocial services for survivors and other members of the community, including male partners. Three areas of focus were identified for future work of FORAL: a need to provide clinical services to women younger than 20 years old in a way that reduces possibility of future stigma, to engage male partners in health education and clinical care and to strengthen linkages with projects addressing psychosocial support and mental health needs of traumatized communities.

This review supports the importance of indigenous expertise in developing strategies and sustainable services in conflict and post-conflict areas. FORAL’s model of engaging CHW, offering health education for all community members, partnering with PHC and hospitals to leverage resources and their principal of avoiding labeling the clinic as one for survivors will help women and their families to comfortably and safely access needed health care services in rural Eastern DRC and other settings that have experienced conflict. This case study does not assess cost-effectiveness. Yet, with limited human resources, equipment, and medication, mobile health services can support and strengthen existing services while reaching populations that are more difficult to access but in need of care as is often true for rural, conflict-affected populations.

## Abbreviations

CHW: Community health workers; DRC: Democratic Republic of Congo; FORAL: Foundation RamaLevina; GBV: Gender based violence; HCP: Health care providers; HIV: Human immunodeficiency virus; NGO: Nongovernmental organization; PHC: Primary health center; PTSD: Post traumatic stress disorder; STI: Sexually transmitted infections; UNDP: United Nations Development Fund.

## Competing interests

The authors declare that they have no competing interests.

## Authors’ contributions

MTM, IZ, BM and RB reviewed clinic activities and designed data management forms. MTM, IZ, BM, RB, PR, OS, AK and NG designed and participated the brief review of clinic activities after one month and extensive evaluation after one year. JM and AK summarized project data from the revised data management forms. AK drafted the manuscript. NG and AK revised the manuscript. All authors read and approved the final manuscript.
